# Identification of genetic molecular markers and immune infiltration characteristics of Alzheimer's disease through weighted gene co-expression network analysis

**DOI:** 10.3389/fneur.2022.947781

**Published:** 2022-08-22

**Authors:** KeFei Duan, Yuan Ma, Jin Tan, Yuyang Miao, Qiang Zhang

**Affiliations:** ^1^Department of Geriatrics, Geriatrics Institute, Tianjin Medical University General Hospital, Tianjin, China; ^2^Department of Urology, Tianjin Institute of Urology, The Second Hospital of Tianjin Medical University, Tianjin, China

**Keywords:** genetic biomarkers, weight gene co-expression network analysis, Alzheimer's disease, immune infiltration, differential genes

## Abstract

**Background:**

Alzheimer's disease (AD) is a progressive neurodegenerative disease that leads to cognitive impairment and memory loss. Currently, the pathogenesis and underlying causative genes of AD remain unclear, and there exists no effective treatment for this disease. This study explored AD-related diagnostic and therapeutic biomarkers from the perspective of immune infiltration by analyzing public data from the NCBI Gene Expression Omnibus database.

**Method:**

In this study, weighted gene co-expression network analysis (WGCNA) was conducted to identify modules and hub genes contributing to AD development. A protein–protein interaction network was constructed when the genes in the modules were enriched and examined by Gene Ontology (GO)/Kyoto Encyclopedia of Genes and Genomes (KEGG) analysis. Furthermore, a gene network was established using topological WGCNA, from which five hub genes were selected. Logistic regression analysis and receiver operating characteristic curve analysis were performed to explore the clinical value of genes in AD diagnosis. The genes in the core module intersected with the hub genes, and four intersection genes (*ATP2A2, ATP6V1D, CAP2*, and *SYNJ1*) were selected. These four genes were enriched by gene set enrichment analysis (GSEA). Finally, an immune infiltration analysis was performed.

**Results:**

The GO/KEGG analysis suggested that genes in the core module played a role in the differentiation and growth of neural cells and in the transmission of neurotransmitters. The GSEA of core genes showed that these four genes were mainly enriched in immune/infection pathways (e.g., cholera infection and *Helicobacter pylori* infection pathways) and other metabolic pathways. An investigation of immune infiltration characteristics revealed that activated mast cells, regulatory T cells, plasma cells, neutrophils, T follicular helper cells, CD8 T cells, resting memory CD4 T cells, and M1 macrophages were the core immune cells contributing to AD progression. qRT-PCR analysis showed that the ATP6V1D is upregulated in AD.

**Conclusion:**

The results of enrichment and immuno-osmotic analyses indicated that immune pathways and immune cells played an important role in the occurrence and development of AD. The selected key genes were used as biomarkers related to the pathogenesis of AD to further explore the pathways and cells, which provided new perspectives on therapeutic targets in AD.

## Introduction

Dementia, a progressive degenerative disease common in older adults, is characterized by a cognitive decline that is severe enough to interfere with daily functioning. Currently, 47 million patients suffer from dementia worldwide, and this number is expected to double by 2050. To date, there exists no cure for any of the causes of dementia (1). The clinical course and medical history remain the main basis for the current diagnosis of dementia. Alzheimer's disease (AD) is the most common cause of dementia. Previously, amyloid (A) aggregates and tau neurofibrillary tangles (NFTs) were the two main pathological features of AD, and were considered as the gold standard for the diagnosis of AD (2). In recent years, the clinical and research application of AD biomarkers has gone through a long process, and people have conducted more sufficient research on Alzheimer's disease and adjusted the diagnosis of Alzheimer's disease. At present, the clinical diagnostic criteria for AD mainly include the IWG-2 criteria developed by the International Working Group (IWG), the NIA-AA criteria formulated by the National Institute on Aging and Alzheimer's Association (NIA-AA), and the “Guidelines for the Diagnosis and Treatment of Alzheimer's Disease in China (2020 version)” released by the Professional Committee on Alzheimer's Disease and Related Diseases of the Chinese Geriatric Health Care Association (Alzheimer's Disease Chinese, ADC). According to the 2011 recommendations of the National Institute on Aging and the Alzheimer's Association on diagnostic guidelines for Alzheimer's disease: Preclinical stages, mild cognitive impairment, and dementia, Alzheimer's disease (AD) is defined by its underlying pathological process and can be recorded by autopsy or biomarkers *in vivo*. In this research framework, the diagnosis is not based on the clinical consequences of the disease (i. e., symptoms/signs), which shifts the definition of AD in living people from a syndrome to a biological structure, focusing on the diagnosis of AD with biomarkers in living people. Cerebrospinal fluid biomarkers, such as Aβ42, T-tau, and P-tau, are recognized as central biomarkers for AD. In addition, the development of new molecules in other pathophysiological pathways that can be used as biomarkers for the diagnosis of AD has made great progress in the last decade. Neuroimaging technology has developed rapidly over the past few decades, with amyloid positron emission tomography (PET) and fluoro-18-2-deoxyglucose PET acting as molecular imaging biomarkers, playing an important auxiliary role in the diagnosis of AD. Amyloid PET radiological tracers can detect the early pathological changes in AD and can visualize and follow up on the pathophysiological changes in patients with AD. Recent studies have proposed a diagnostic algorithm to comprehensively analyze the optimal time points, amyloid proteins of PET biomarkers, tau, and genetic markers for the early diagnosis of AD, enabling a comprehensive study of the pathogenesis of the disease (3). In 2021, the guideline was further revised, and the diagnosis of AD included more biomarkers, such as the plasma a β 42/A β 40, P - tau217, P - tau181, and NFL levels and cerebrospinal fluid a β 42/A β 42/A β 40. P - tau181, P - tau217, t - tau, and NFL levels, which can be used for the early diagnosis of AD-derived MCI and the evaluation of disease progression. The diagnosis of AD through neuroimaging is also more perfect. NIA-AA diagnostic criteria (2011, 2018) and iwg-2 (2014) are adopted to pay attention to the application of AD-related biomarkers in the diagnosis of AD-derived MCI (4).

Research in the field of AD has rapidly progressed over the past few decades. Recent research suggests that the risk of AD is partly driven by genetic factors. In 2019, a large-scale genome-wide association meta-analysis identified 25 genome-wide loci, some of which were identified as familial proxies for AD or dementia and were involved in tau binding, amyloid precursor metabolism, immunity, and lipid metabolism (5). Apolipoprotein E4 (APOE4) has been reported to influence AD, in part through its immunoregulatory functions. Additionally, APOE4 variants are the largest genetic risk factors for AD. This function of APOE may be related to the triggering receptor expressed on myeloid cells 2 (TREM2), which is expressed by the microglia in the central nervous system (CNS) (6). TREM2 not only affects the microglial function in amyloid and tau pathologies but also participates in inflammatory responses and metabolism, acting alone or in combination with other molecules (e.g., APOE) (7). Relevant literature has reported on TREM2 and PLCγ2, as well as the potential role of protein kinase C (PKC) and PKC regulators as new therapies for neuroinflammation and neurodegenerative diseases in promoting the activation of repair/regeneration microglia subtypes (8). Neuroinflammation-induced neurodegeneration and immune cell infiltration are the two hallmarks of AD (9).

As is well-known, neuroinflammation is the main factor for AD progression. Immune cell infiltration is a type of neuroinflammation that causes extensive damage to the neurons in the CNS *via* the interaction between various immune system cells and CNS neurons. AD has pro-inflammatory properties that can activate peripheral leukocytes and transfer them to the center, damaging the CNS (10). In early AD, activated neutrophils accumulate in the blood, migrate to the CNS, promote local inflammation, and cause progressive damage to the blood–brain barrier (BBB). The BBB integrity is impaired, and fibrinogen penetration through the leaky BBB causes various abnormalities, including persistent fibrin aggregation and coagulation, microglia-mediated production of reactive oxygen species, and neurons and synapses. The vitality of the contact connection is reduced (11). Related studies have shown that T cells play an important role in promoting AD development. After the onset of AD, T cells infiltrate the CNS and secrete pro-inflammatory mediators, such as CD8 T cells (12); additionally, they may directly act on the neurons that regulate synaptic function, leading to dysfunction. T cells can cooperate with other cells to cause AD formation. On the other hand, activated B cells, which have been extensively studied over recent years, gradually accumulate and infiltrate into the brain parenchyma after the onset of AD, resulting in Aβ protein deposition (13). Neutrophils also play a role in AD progression; by using microPET to detect tracer uptake at different stages of AD, a previous study revealed that the neutrophil activity was increased in the brain and heart of AD models and that infiltrating neutrophils could induce the microglia *via* CAP37 release (14). Immune cells and their induced inflammatory responses can have detrimental effects on the immune microenvironment of the CNS, thereby exacerbating AD progression (8). Nevertheless, the mechanisms of action of these immune cells in AD have not been thoroughly investigated (15). Therefore, a systematic approach is urgently needed to explore the correlation between immune-infiltrating cells and AD in more detail.

Alzheimer's disease (AD) is a neurodegenerative brain disease characterized by extracellular amyloid plaques and neurofibrillary tangles in the brain, which affect different areas of the brain based on the different progression of AD disease (16). Lesions early in AD progression originate from the entorhinal cortex (EC) and the hippocampus (HIP) (17), and as AD evolves, neurofibrillary tangles develop from layer II of the entorhinal cortex (EC-II) to the limbic and associated cortices (18). The hippocampus is one of the involved regions, and its atrophy is a widely used biomarker in the diagnosis of AD (19). The hippocampus is a brain region critical for learning and memory, and is particularly vulnerable to damage in the early stages of Alzheimer's disease (AD). Emerging evidence suggests that alterations in adult hippocampal neurogenesis represent early key events during AD. From a functional perspective, hippocampal neurogenesis plays an important role in structural plasticity and network maintenance (20). At present research, hippocampal tissue is the best sample source for studying the mechanism of learning and memory function in Alzheimer's disease (AD) patients and healthy controls (21). Many mechanisms are related to lesions in the hippocampal region after AD lesions. For example, the accumulation of mAPP and A causes abnormal mitochondrial, synaptic, and autophagy/mitophagy abnormalities in hippocampal neurons, leading to neuronal dysfunction (21). Wang's team reported that neuronal miR-124 is significantly increased in the hippocampus of TG2576 mice, and that miR-124/PTPN1 pathway is a key mediator of synaptic dysfunction and memory loss in AD, thus miR-124 / PTPN1 pathway can be considered as a promising new therapeutic target for patients with AD (22). Genes encoding the members of the m6A methyltransferase METTL3 and RBM15B, the m6A methyltransferase complex (MACOM), are downregulated and upregulated in the hippocampus, respectively. The aberrant expression and distribution of METTL3 in AD mind may represent surface transcription as a mechanism of altered gene expression patterns associated with disease pathogenesis (23). The literature has reported that the injection of BM-MSCs at the hippocampus site in the brain can improve the cognitive impairment in AD model mice by improving astrocytic inflammation and synaptogenesis. It has been confirmed that the exosomal transfer of miR-146a is involved in the correction of cognitive impairment in AD model mice (24). In conclusion, with the progression of the disease after AD, the whole brain region was abnormal, among which the neurogenesis in the hippocampal region was the most significant. We selected the hippocampal region for a detailed study and analyzed the RNA changes in this region to provide a basis for finding the target gene of AD and further extracellular vesicle drug therapy.

Weighted gene co-expression network analysis (WGCNA) is an effective method for demonstrating the correlation of gene expression using a microarray database (25). WGCNA is used to identify correlated genes (modules), analyze the characteristic genes of modules or the hub genes that determine modules of interest based on the correlation coefficients between modules and phenotypic traits, and explore the relationship between modules and traits. This method is commonly applied to explore genetic markers and therapeutic targets in various diseases (26). WGCNA is widely utilized to study various diseases, such as diabetes (27) and cancer (28), and is also applied to brain imaging data analysis (29), and proteomic and metabolomic analyses (30).

Several previous molecular biology studies have shown that immune cells and their induced inflammatory responses can have long-term detrimental effects on the immune microenvironment of the CNS, thereby exacerbating AD progression (26). Nonetheless, immune-infiltrating cells and immune-related genes associated with AD remain unknown. Therefore, the present study aimed to identify immune-infiltrating cells and related genes in AD and to investigate their biological characteristics and involved pathways in order to provide promising targets for further studies.

## Materials and methods

### Data collection and preprocessing

**Figure 1:**
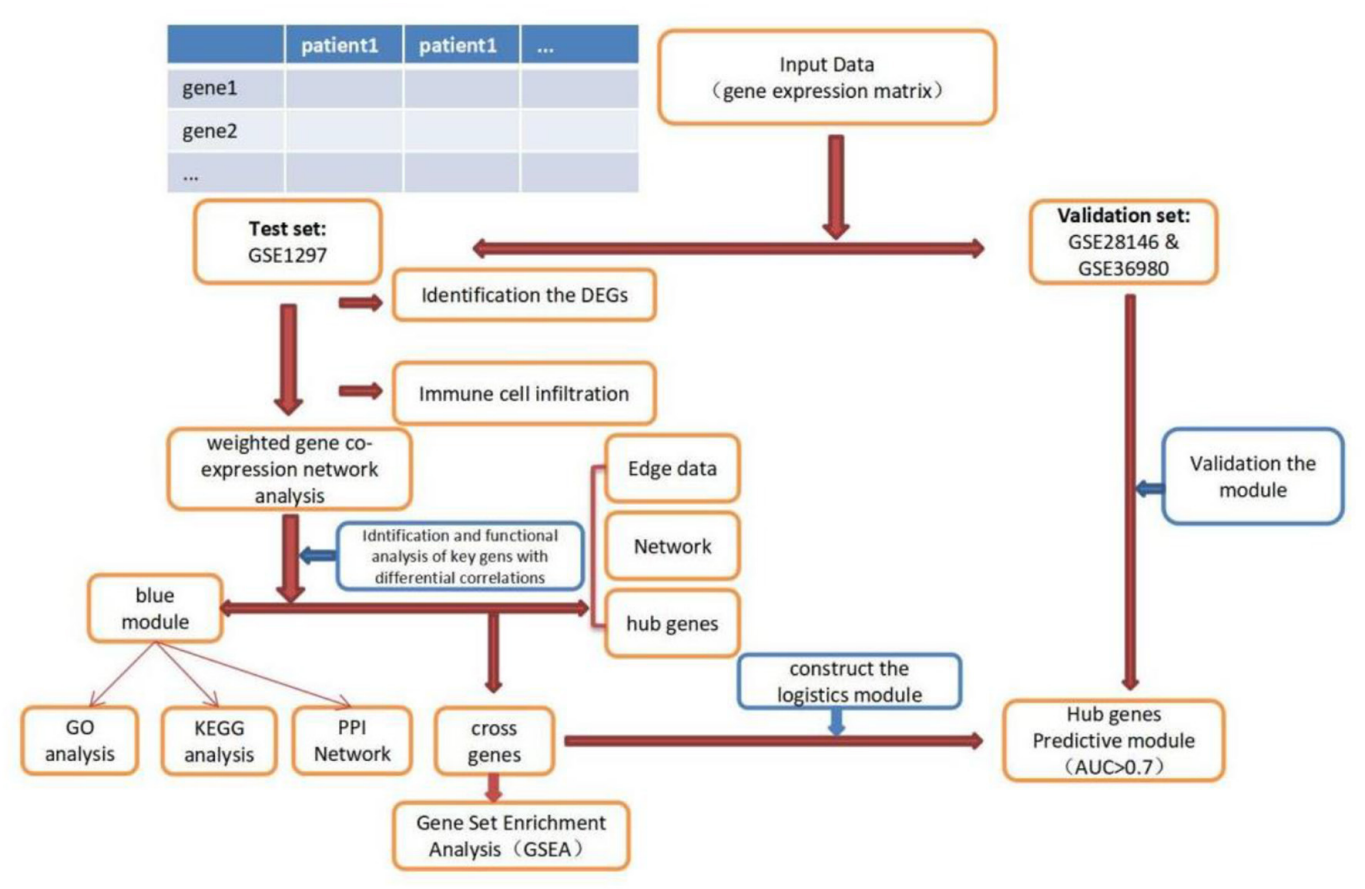
Flowchart of the study. DEGs, differential expression genes; GO, Gene Ontology; KEGG, Kyoto Encyclopedia of Genes and Genomes; PPI, protein-protein interaction; GSEA, Gene Sets Enrichment Analysis.

The expression profile datasets of human Alzheimer's disease patients and controls were retrieved from the comprehensive gene expression database (GEO) for analysis. Select the data with the type of expression profiling by array for detailed analysis. The following datasets were selected for analysis in this study: GSE1297 (22 AD samples and 9 normal control samples from the hippocampus, Blalock EM et al., published 2004), GSE28146 (22 AD samples and 8 normal control samples from the hippocampus, Blalock EM et al., published 2011), and GSE36980 (8 AD samples and 10 normal control samples from the hippocampus, Nakabeppu Y et al., published 2013). Further details are provided in Table 1. The GSE1297 dataset was selected as the train set to explore the relevant modules and hub genes, whereas the other datasets were used as validation sets, from which the GSE28146 was selected as the validation set 1, and the GSE36980 was selected as the validation set 2 (31). The GSE1297 dataset was analyzed using the GPL196 chip analyzer platform (32). Data on related clinical characteristics, including age, disease status, postmortem interval, NFTs, Braak stage, Mini-Mental State Exam (MMSE) score, and sex, were also retrieved. All analyses were conducted using R software version 4.1.5 (R Foundation for Statistical Computing, Vienna, Austria). A flowchart illustrating the study design is shown in Figure 1. Samples for the test and validation sets were obtained from the hippocampus.

**Table 1:** The details of each datasets, sample source, Number of samples.

**Datasets**	**Brain region**	**Sample size of control group**	**Sample size of AD group**
GSE1297	Hippocampus	9	22
GSE36980	Hippocampus	10	8
GSE28146	Hippocampus	8	22

#### Code availability

All codes used in this manuscript are available on github at Comparing main...Duankefei-patch-1-1 · Duankefei/AD-WGCNA (github.com).

### Analysis of differentially expressed genes

**Figure 2:**
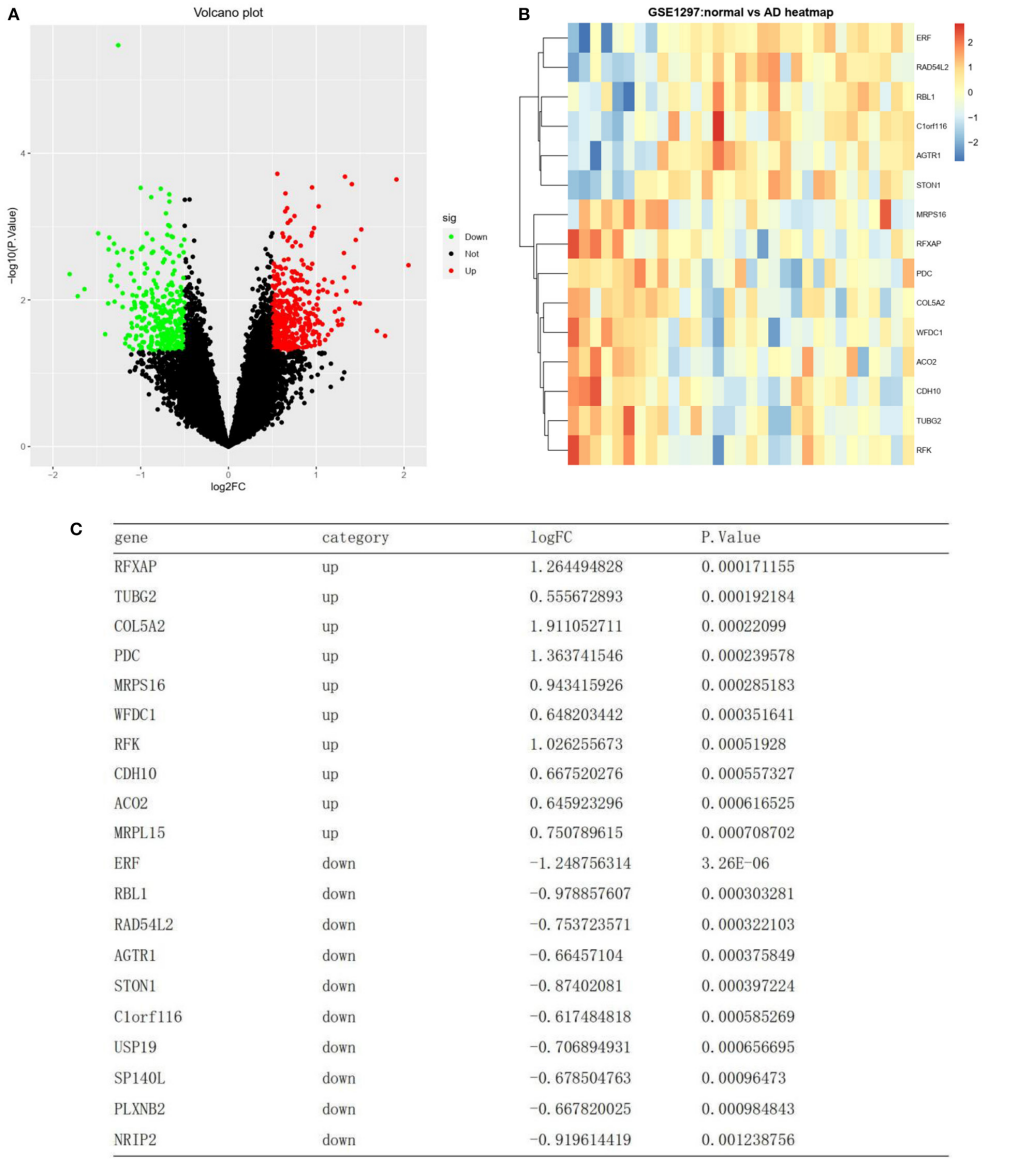
Differentially expressed RNAs. **(A)** A volcano plot showing the expression level of 4440 mRNAs. Green, and red dots represent the top 50 down-regulated and up-regulated mRNAs, respectively. Black dots represent not differentially expressed. **(B)** Heat map clustering of the differentially expressed genes between AD and normal samples. **(C)** List of the top 10 most significant up and down regulated genes.

Differential analysis was carried out on the training set GSE1297 to screen differential genes. Based on the annotation platform of each expression profile, the probe was combined with the gene name to remove the empty probe. The lncRNA transcript was deleted from the dataset. A total of 4,440 genes were selected for differential expression gene (DEG) analysis. DEG analysis was conducted using the “limma” R package, and the expression data of the gene matrix were transformed using log2. The *P*-values were adjusted using the false discovery rate (FDR) method, and genes with abs |log2 fold change| ≥0.5 with a *P*-value of <0.05 were screened as DEGs. Upregulated and downregulated DEGs were screened according to the | log2-fold change | and plotted as volcano plots using the “ggplot2” R package (Figure 2A). DEGs were presented as a heatmap using the “pheatmap” R package (Figure 2B).

### Weighted gene co-expression network analysis

The “WGCNA” R package was used for WGCNA of 4,440 selected genes to explore modules related to clinical features. All samples were included in the group, and network topology analysis was performed with a threshold cutoff ranging from 1 to 20. An appropriate soft power threshold was selected to calculate the adjacency matrix. The power value of the predicted gene co-expression network was 5 (scale-free *R*^2^ = 0.85), indicating a scale-free topology with complete module characteristics. A dendrogram was generated, and similar clinical traits of the samples were explored to determine whether the 31 samples in the GSE1297 dataset were suitable for network analysis. The connectivity of the model was calculated, a connection matrix was constructed, and the number of modules was adjusted.

The soft power threshold was calculated, the adjacency matrix was converted into a topological overlap matrix (TOM), and hierarchical clustering was constructed according to the TOM phase-difference matrix (1-TOM). The gene expression and clinical features were analyzed. Gene significance and modular signature genes (MEs) were determined, and the relationship between MEs and clinical features of AD was analyzed using Pearson's correlation coefficients. The function for *P*-values by Student's *t-*test was used to determine the Student's asymptotic *P*-value. Except for the gray module, the most significant *P*-value in the AD status column was selected as the key module (*P* < 0.05). Genes in the key module were selected for subsequent analysis.

### Functional enrichment analysis and protein–protein interaction network analysis of Alzheimer's disease-associated module

Each module is a functional unit (sub-network) with a distinct biological function that we try to find out by building modules using WGCNA. In order to explore the biological significance of the selected module (blue module, significantly correlated with Alzheimer's disease) genes, Gene Ontology (GO) annotation and Kyoto Encyclopedia of Genes and Genomes (KEGG) enrichment analysis were performed using the DAVID tool (http://david.abcc.ncifcrf.gov/). The GO terms and KEGG pathways were visualized using the “ggplot” R package.

The PPI plots are a great way to extrapolate the gene expression of mRNA-based findings to the protein level. Potential interactions among proteins encoded by these genes were predicted using the STRING web server (version 11.5; http://string-db.org). A combined interaction score of >0.5 was considered to be statistically significant. The minimum required interaction score was set at the highest confidence level (0.900). The PPI network was visualized using the Cytoscape software (version 3.8.2).

### Network construction and identification of the hub genes

The edge node file for Alzheimer's disease exported from WGCNA was imported into Cytoscape software, and a network was constructed based on the edge with a weight >0.7, for visualization of the module gene–gene connections. Hub genes in Alzheimer's disease were calculated using 12 analysis algorithms of the CytoHubba plugin in Cytoscape, which are MCC, DMNC, MNC, Degree, EPC, BottleNeck, EcCentricity, Closeness, Radiality, Betweenness, Stress, Clustering, and Coefficient. The top five genes in the network were selected and presented as hub genes (Cytoscape software version 3.6.2; cytoHubba plug-in). The 12 algorithms are the internal programs of cytohubba, and the algorithm sorts the calculated values from large to small according to the results.

### Efficacy evaluation: Predictive model with hub genes in Alzheimer's disease and model validation

A prediction model was constructed by the logistic regression analysis. A receiver operating characteristic (ROC) curve was drawn using the “ROCR” R package, and the area under the curve (AUC) was calculated to investigate the clinical value of each gene in diagnosing AD. The “ROCR” R package was used to evaluate and visualize the ability of hub genes in Alzheimer's disease in distinguishing disease states (classifier performance). The AUC values > 0.7 indicated the significant specificity and sensitivity of the model. AUC values closer to 1 indicate a better model or classifier performance. The classifier performance of two validation datasets (GSE28146 and GSE36980) was used to corroborate the performance of the training dataset.

### Gene set enrichment analysis

Intersection genes between hub genes and the key modules were selected for further analysis. Specific signaling pathways associated with hub genes and potential molecular mechanisms modulating AD progression were explored. Gene set enrichment analysis (GSEA) was used to explore if the list of hub genes in Alzheimer's disease contained (significantly enriched with) any GSEA gene sets. GSEA gene sets are functionally related groups of genes, usually in the same pathway, created based on curated knowledge present in the GSEA database. GSEA gene sets that showed an opposite correlation in the two states (Alzheimer's disease and Control) were considered significant. The possible biological functions of key genes were explored by GSEA (3.0.0 Edition, Broad College, MIT, and University of California Board of Trustees). The “c2.cp.kegg.v3.0.symbols” function was used for enrichment analysis, and a consensus *P*-value was calculated for each genome using significant GSEA results. The default weighted enrichment method was used for enrichment analysis. The random combination is set to 1,000 times. The high and low expression levels of the hub gene were enriched and analyzed by GSEA analysis. Pathways with *P*-values of <0.05 and FDR <0.25 were screened.

### Immune cell infiltration analysis

Different immune cell types in AD brain tissues were analyzed by calculation using CIBERSORT. Expression data were combined, and immune cell infiltration was determined (*P* < 0.05). The percentage of immune cell types was calculated and represented as bars. A heatmap of 22 immune cells was generated using the “pheatmap” R package, and a correlation heatmap showing the relationship among 22 types of infiltrating immune cells was generated using the “corrplot” package.

### Validation of the expression level of screened hub-mRNAs in AD by qRT-PCR

Construction of AD cell model: HT22 cells were exposed to 10 μM of A β protein for 24 h. Total RNA (Invitrogen, China) was extracted from the AD model and a normal HT22 cell line with Trizol reagent according to the manufacturer's instructions. Total RNA was extracted from cell lines using TRIzol reagent (Invitrogen, China) according to the manufacturer's protocol. Reverse transcription was conducted on RNA to produce cDNA using Revert Aid First Strand cDNA Synthesis Kit (ThermoScientific; United States) according to the manufacturer's guidelines. Quantitative reverse transcription–polymerase chain reaction (qRT–PCR) was performed using FastStart Universal SYBR Green Master (ROX) (Roche; United States). GAPDH was used as the internal control, and the relative expression levels of mRNA were calculated by the 2–ΔΔCt method. The primer sequences utilized in this study were as follows: GAPDH-F: AGGTCATCACTATTGGCAACGAG, GAPDH-R: TTGGCATAGAGGTCTTTACGGAT; ATP2A2-F: AAGACAGGCACACTTACCACAAACC, ATP2A2-R: GGCACTTCACTGGCTTATCATCC; ATP6V1D-F: TGCTGATGGGTGAAGTGATGAG, ATP6V1D-R: TGCTGAAGTCCCCTGCTGTG; CAP2-F: CCAACAACCCCAAGAGAATGAAG, CAP2-R: CGATGCTTTCACTGACTGCCG; and SYNJ1-F: CCCATCGTGTTCGTATGTCAAG, SYNJ1-R: TATCAGAAGCGTGTTCAGAGGC.

### Statistical analysis

The *T*-test was used for measurement of the data (expressed as a mean ± SEM), while the chi-squared test was used for categorical data (presented as percentages). The logistic regression algorithm was used to build the predictive model. All statistical analyses were performed by R software version 4.1.5 (R Foundation for Statistical Computing, Vienna, Austria) and GraphPad Prism 8. All experiments were done in triplicate. Significance was defined as *P* < 0.05 for two-sided tests.

## Results

### Identification of DEGs in normal and AD samples

A total of 4,440 genes were selected after preprocessing, and 756 DEGs were identified between the AD and normal samples. Of these 756 DEGs, 442 were upregulated, while 314 were downregulated (top 50 upregulated and top 50 downregulated genes in | log2FC |) (Figure 2A). A heatmap showing DEG clustering is presented in Figure 2B. The results indicated that the AD and control samples could be significantly distinguished on the basis of DEG characteristics. The top 10 most significant up- and downregulated genes are listed in Figure 2C.

### Identification of gene co-expression modules

**Figure 3:**
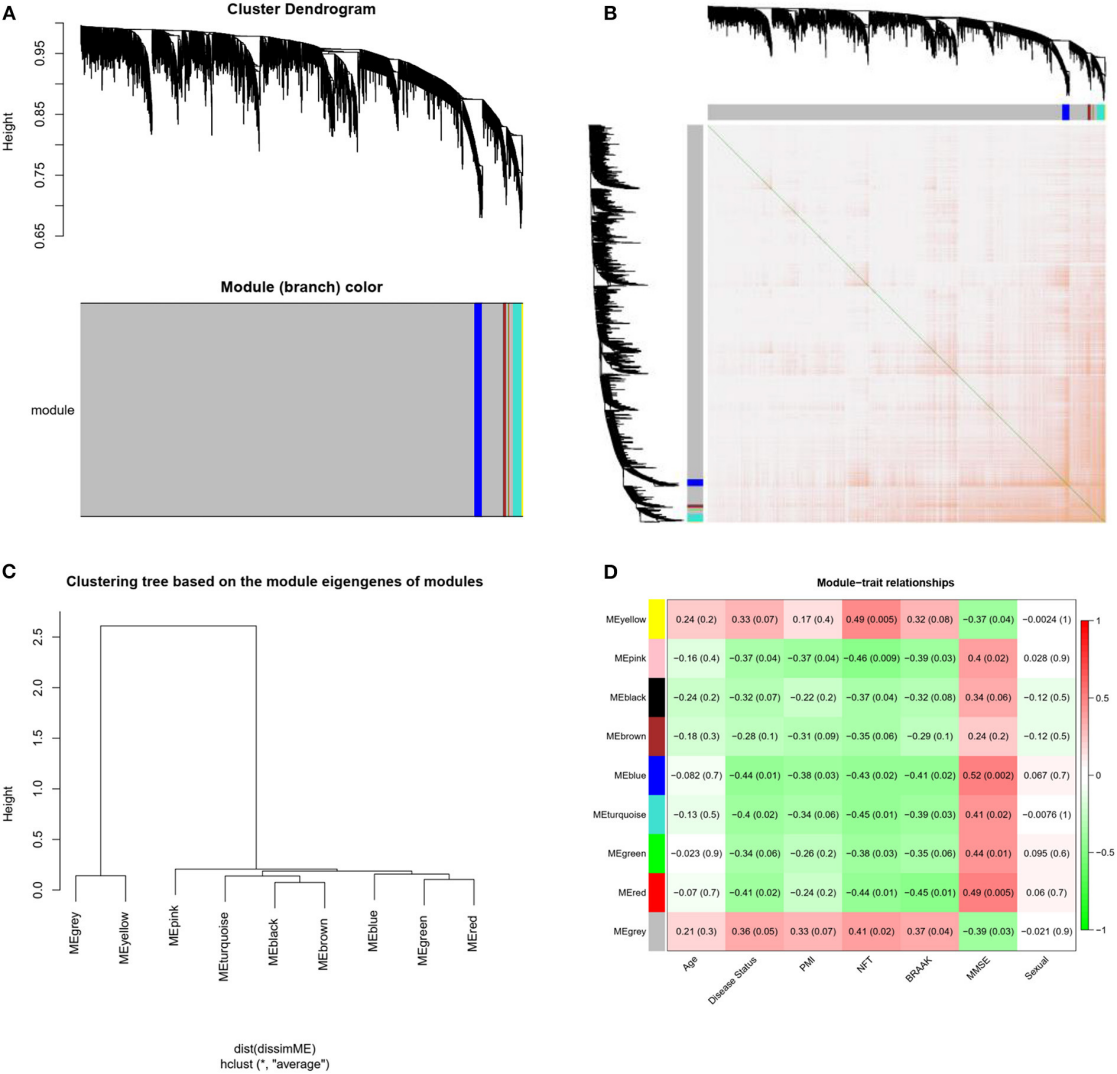
Modules related to the clinical traits of AD. **(A)** Cluster dendrogram of genes: Heatmap of co-expressed genes. Different colors shown on X and Y axes denote different modules. **(B)** Heat map of co-expressed genes. Different colors on X and Y axes represent different modules. The depth of yellow color indicates the degree of connection between modules. **(C)** Dendrogram of Module Eigengenes(MEs) obtained through WGCNA. **(D)** Correlation between modules and traits. Red and blue represents high adjacency and low adjacency, respectively. PMI, postmortem interval; NFT, neurofibrillary tangle; MMSE, mini-mental state examination.

Weighted gene co-expression network analysis was conducted on the expression data of 4,440 mRNAs in the GSE1297 dataset. The “picksoft Threshold” function in the WGCNA software package was used to filter out the power parameters within the range of 1–20. The soft power threshold was set at 5 (scale-free *R*^2^ = 0.85) to obtain a scale-free topology network and analyze reliability. The cutoff height was set at 0.2, and clustering was merged to produce nine modules (Figure 3A). The TOM and dissTOM = 1 – TOM were obtained with non-clustering DEGs in the gray module (Figure 3B). MEs were used as illustrative profiles, and module correspondence was computed using eigengene correlation. The interaction relationship between the nine modules was determined, and a network heatmap was generated (Figure 3C). The heatmap for module trait correlation showed that the most significant correlation feature was in the blue module, which exhibited a significant negative correlation with the MMSE score (correlation coefficient = −0.52, *P* = 0.002; Figure 3D). This finding indicated that the blue module (correlation coefficient = 0.44, *P* = 0.01; Figure 3C) was the key module related to the AD status.

### Significant enrichment of genes related to AD and PPI network analysis of the key module

**Figure 4:**
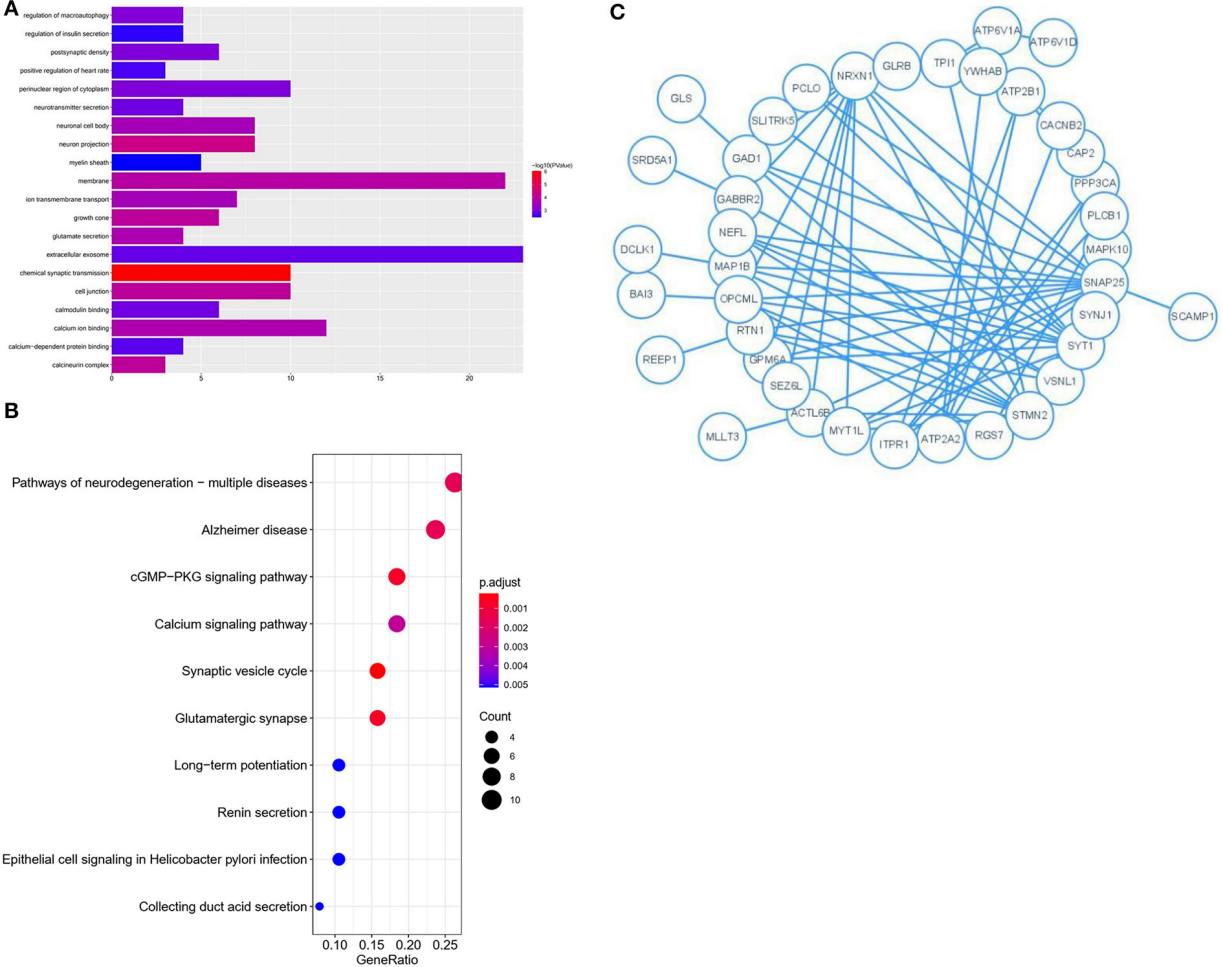
GO and KEGG analysis for genes co-expressed with mRNAs in Blue modules. **(A)** Bar plot of the top 20 enriched GO terms. **(B)** Bubble chart of the top 20 enriched KEGG pathways. **(C)** Module analysis of the PPI network.

The 76 genes identified in the blue module were analyzed in detail. GO and KEGG enrichment analysis was performed to explore the pathways involved in the genes in the key module. The top 20 significantly enriched GO terms were retrieved from the GO functional annotation (Figure 4A). Functional enrichment analysis revealed that genes were significantly enriched in biological process terms (positive regulation of heart rate, glutamate secretion, regulation of macroautophagy, neurotransmitter secretion, regulation of insulin secretion, ion transmembrane transport, and chemical synaptic transmission), cell component terms (calcineurin complex, myelin sheath, growth cone, postsynaptic density, neuron projection, neuronal cell body, cell junction, perinuclear region of cytoplasm, membrane, and extracellular exosome), and molecular function terms (calcium-dependent protein binding, calmodulin binding, and calcium ion binding). The KEGG pathway analysis showed that the genes were mainly enriched in the synaptic vesicle cycle, cGMP-PKG signaling pathway, glutamatergic synapse, pathways of neurodegeneration, multiple diseases, and AD. The top 20 KEGG pathways were selected for further analysis (Figure 4B). The Y-axis of Figures 4A,B shows the biological functional pathways through enrichment analysis, and the X-axis represents the number of genes enriched by each biological functional pathway. The GO and KEGG analysis indicated that the genes in the key module were enriched during neural cell differentiation and growth, as well as during neurotransmitter delivery, some of which are involved in the pathogenesis of neurodegenerative diseases or Parkinson's disease.

Genes identified in the blue module were selected for further analysis. A PPI network comprising 76 nodes and 28 edges was constructed using the STRING tool (https://cn.string-db.org/) to further determine their associations at the protein level and visualize the PPI network (Figure 4C; Cytoscape software). The network status was as follows: average node degree = 0.737 and expected number of edges = 9.

### Identification and efficacy evaluation of hub genes

**Figure 5:**
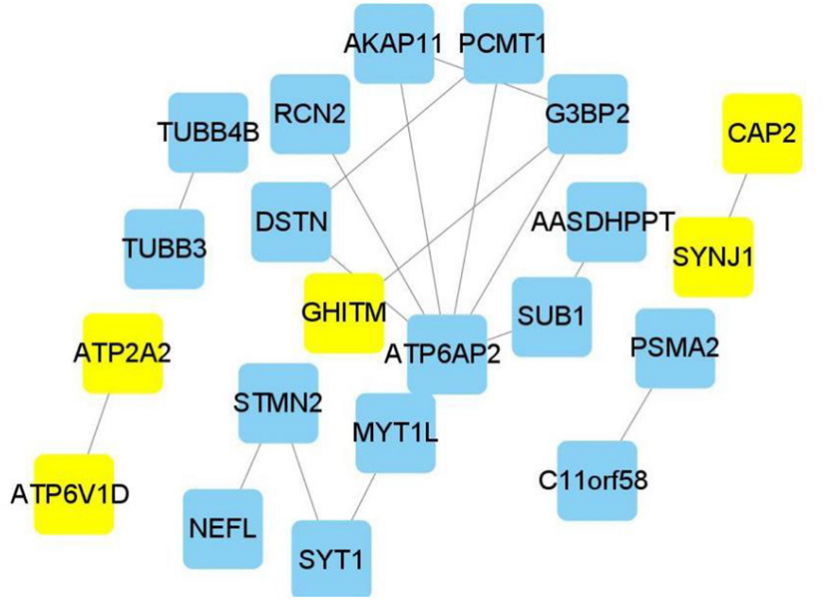
Hub genes in Alzheimer's disease module. Construction of a network and hub genes. Hub genes highlighted in Cytoscape module visualization. The network yellow color box represents hub genes. The lines indicate correlation between genes.

Edge data calculated from WGCNA were entered into the Cytoscape software, and the weight of network construction was set at >0.7 (Figure 5). The cytoHubba plug-in of the Cytoscape software was used to comprehensively analyze the results of 12 algorithms. The following top five genes were selected as the hub genes: *ATP2A2, ATP6V1D, CAP2, SYNJ1*, and *GHITM*.

A prediction model for the logistic regression analysis was constructed. The regression equation was as follows: Y = *62.353420* + *3.620291* ^*^ *ATP2A2-16.089225* ^*^ *ATP6V1D* + *2.823338* ^*^ *CAP2* + *2.035213* ^*^ *SYNJ1* + *1.501149* ^*^ *GHITM*.

**Figure 6:**
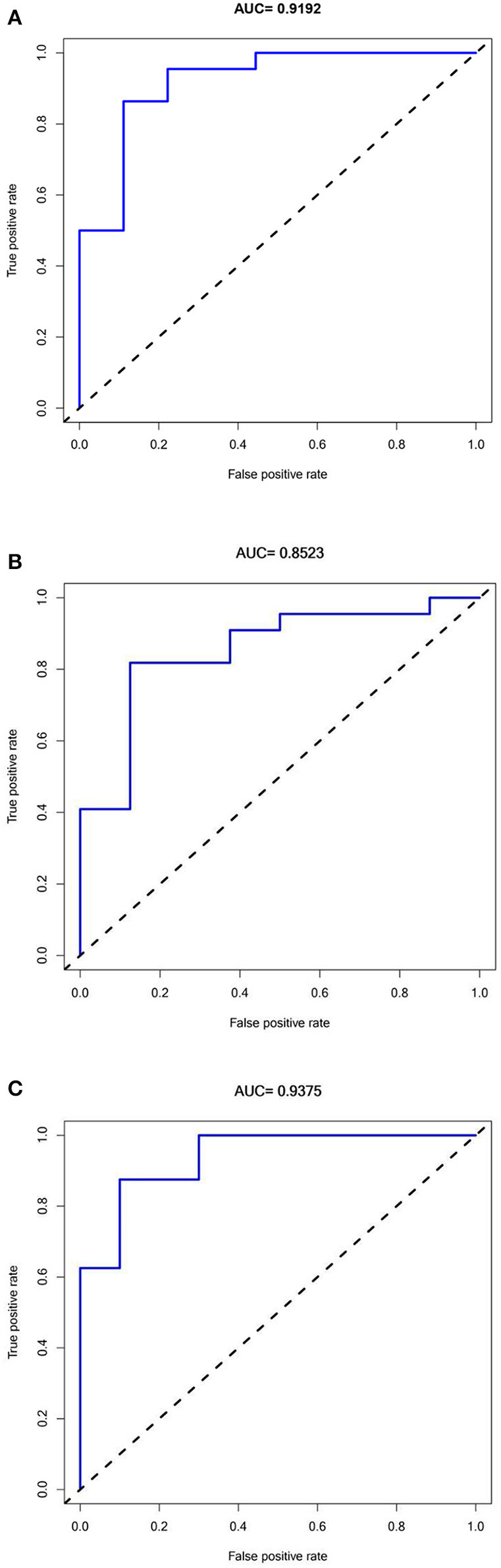
Receiver Operator Characteristics curves for Predictive modeling with Hub genes in Alzheimer's disease. **(A)** Training dataset GSE1297:AUC = 0.9192. **(B)** Validation dataset 1 GSE28146: AUC = 0.8523. **(C)** Validation dataset 2 GSE36980: AUC =0.9375.

A ROC analysis of the prediction model comprising five hub genes was performed using the GSE1297 dataset as the test set, and an AUC value of 0.9192 (Figure 6A) was obtained. The model was further validated using the verification sets (GSE28146 and GSE36980), obtaining AUC values of 0.8523 and 0.9375 (Figures 6B,C), respectively. All AUC values in the test and validation sets were >0.7, indicating that the model had high sensitivity and specificity. Furthermore, this finding suggested that the five hub genes were potential biomarkers for detecting AD.

### Potential biological functions on GSEA

Considering the intersection of hub genes and blue module genes, four cross genes were obtained, namely, *ATP6V1D, CAP2, SYNJ1*, and *ATP2A2*. The intersection genes were further analyzed. Further analysis of the biological pathways revealed that hub genes were implicated in immune, inflammatory, and other metabolic processes. The samples in the GSE1297 dataset were assigned to high-expression and low-expression groups according to the expression levels of *ATP2A2, ATP6V1D, CAP2*, and *SYNJ1*. The four hub genes corresponded to 10, 8, 9, and 8 enrichment pathways, respectively, in the high-expression group.

**Figure 7:**
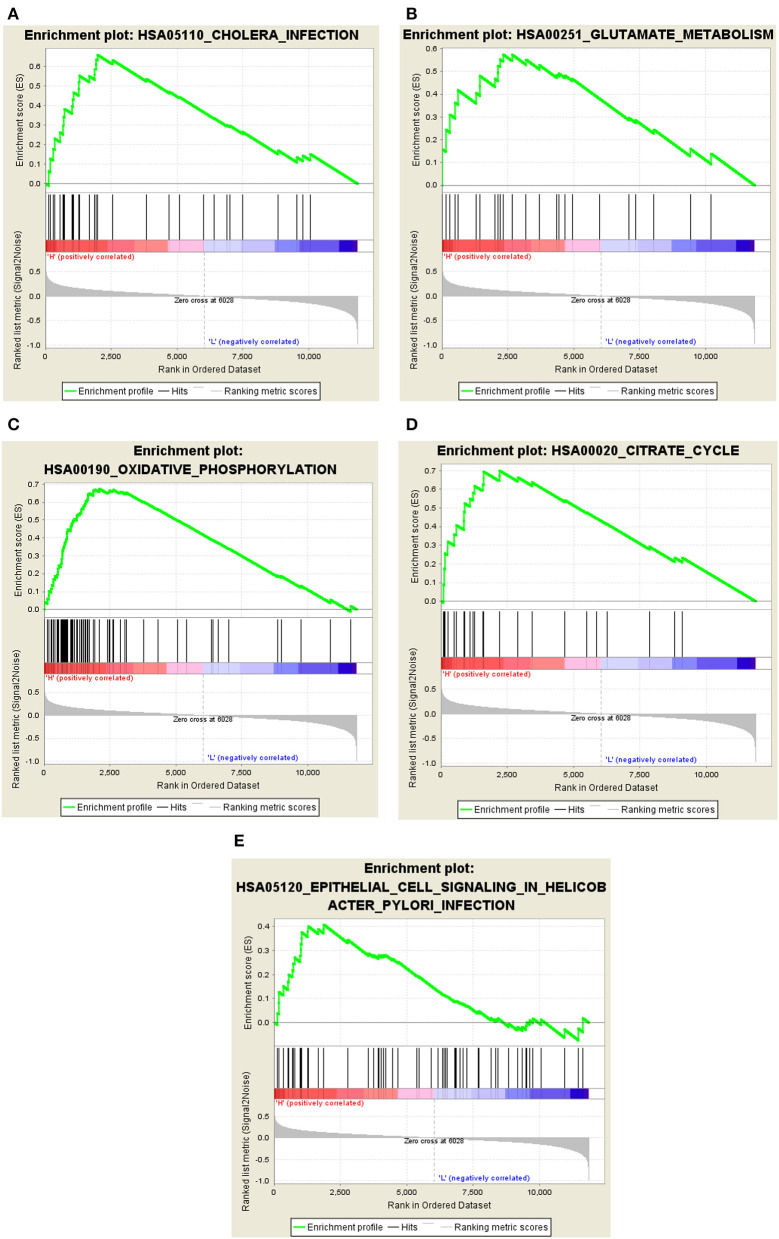
Significant GSEA gene sets in core genes to AD. Results of Gene set enrichment analysis for the four genes (GSEA, www.broadinstitute.org/gsea/,KEGG pathways). **(A)** Cholera infection. **(B)** Glutamate metabolism. **(C)** Oxidative phosphorylation. **(D)** Citrate cycle. **(E)** Epithelial cell signaling in *Helicobacter pylori* infection.

The GSEA results indicated that cholera infection, oxidative phosphorylation, citric acid cycle, glutamate metabolism, ubiquitin-mediated proteolysis, cell signaling, alanine and aspartate metabolism, RNA polymerase, epithelium, proteome, pyruvate metabolism, and selenium amino acid metabolism pathway in *Helicobacter pylori* infections were significantly enriched in the high-expression group of hub genes (*P* < 0.05) (Figures 7A–E). Immune/infection pathways, such as cholera and *H. pylori* infection pathways, were crucial for the high expression of AD. The number of pathways associated with low gene expression was significantly reduced, including the metabolism of cytochrome P450 to exogenous substances, cytokine–cytokine receptor interactions, porphyrin and chlorophyll metabolism, mature diabetes in young people, and cellular communication. A detailed analysis of core gene enrichment pathways should be conducted in further studies to explore the pathogenesis of AD, and the specific mechanism should be verified by *in vitro* and *in vivo* studies.

### Immune cell infiltration analysis

**Figure 8:**
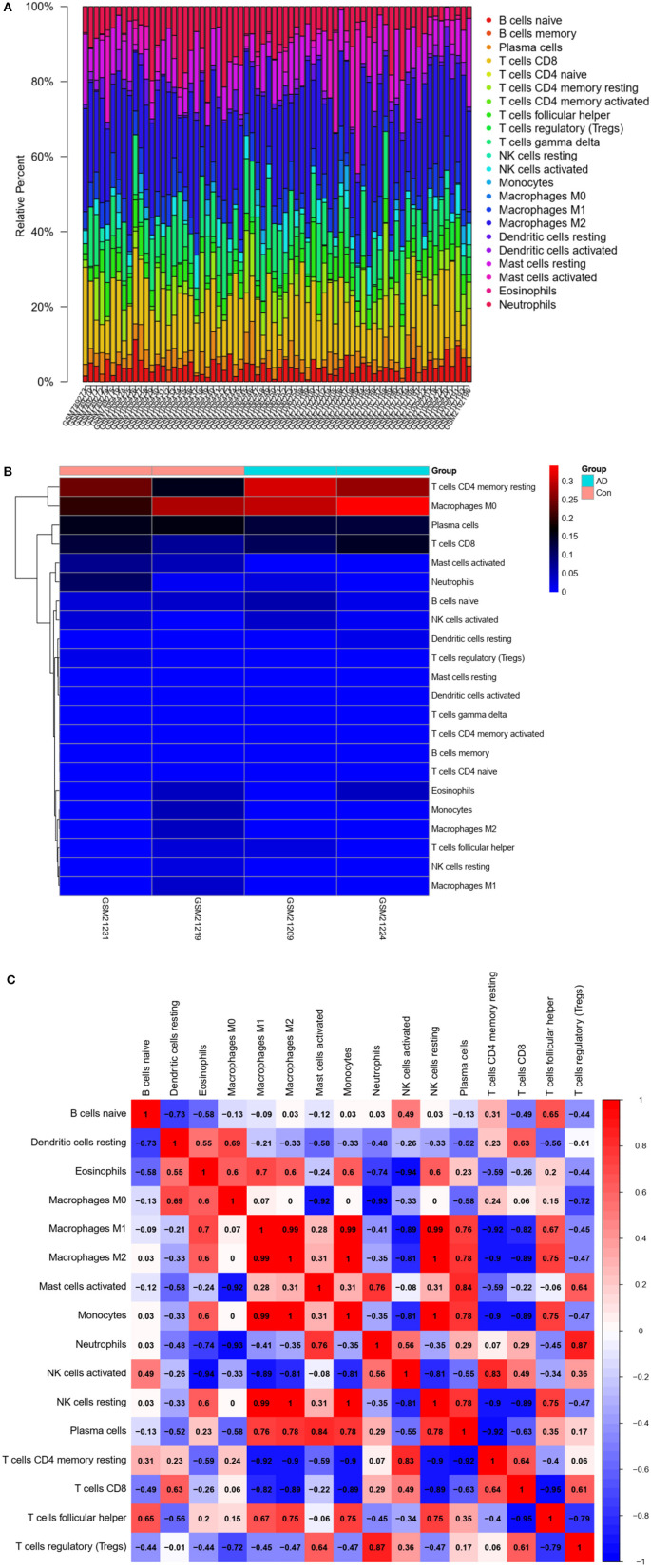
Immune infiltration (immune cell type gene enrichment) in Alzheimer's disease. **(A)** The relative percentage of 22 types of immune cells: Percentage of immune cell types in Alzheimer's disease and Control. **(B)** A heat map of 22 types of immune cells: Correlation between 22 types of immune cells using their expression data in CIBERSORT tool's database. **(C)** Distribution and visualization of immune cell infiltration. Correlation matrix showing the composition of all 22 immune cell subtypes. Horizontal and vertical axes represent immune cell subtypes. High, low, and unchanged immune cell subtype compositions are shown in red, blue, and white, respectively.

The CIBERSORT algorithm was used to predict immune cell infiltration in AD patients and controls. The percentage of 22 immune cells in each sample was presented using a bar graph, as shown in Figure 8A. Samples with significant immune infiltration (*P* < 0.05) were selected, and a heatmap showing the percentage of immune cells was generated (Figure 8B). Correlation analysis of 22 immune cells revealed a positive correlation between regulatory T-cell infiltration and neutrophil infiltration (*r* = 0.87), a positive correlation between plasma cells and activated mast cells (*r* = 0.84), a negative correlation between T follicular helper cells and CD8 T cells (*r* = −0.95), a negative correlation between resting memory CD4 T cells and M1 macrophages (*r* = −0.92), and a negative correlation between CD8 T cells and M1 macrophages (*r* = −0.82) (Figure 8C). A significant difference in immune cell infiltration was observed between the brain tissues of AD patients and normal brain tissues. These findings indicated that regulatory T cells, neutrophils, plasma cells, activated mast cells, T follicular helper cells, CD8 T cells, resting memory CD4 T cells, and M1 macrophages were potential core immune cells involved in the promotion of AD progression.

### The experiment of hub genes

**Figure 9:**
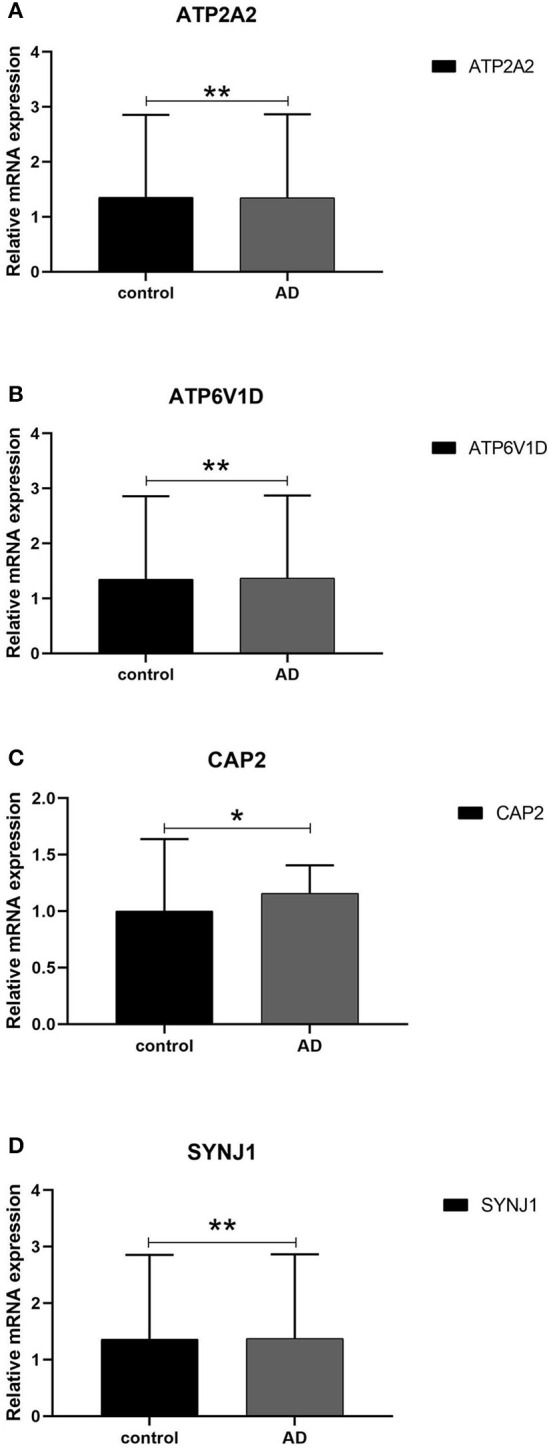
qRT -PCR validation of the hub gene between AD and normal controls. **(A–D)** All experiments were carried out three times, and the data were expressed as mean±SEM (*p < 0.05, **p < 0.01, ns, no significance).

We next conducted qRT-PCR experiments to detect the relative expression level of hub genes in the AD model group and normal HT22 cell group. The data showed that there were significant differences in the mRNA expression levels of ATP2A2, ATP6V1D, CAP2, and SYNJ1 between the AD model group and the normal HT22 cell group (*P* < 0.05, ATP2A2; *P* < 0.05, ATP6V1D; *P* < 0.01, CAP2; *P* < 0.05, SYNJ1) (Figure 9). These identified hub genes might function as the potential diagnostic and prognostic biomarkers.

## Discussion

Alzheimer's disease is influenced by genes, environment, and aging and is clinically characterized by progressive impairment in cognitive domains, such as memory and space. Over recent years, clinical history and disease course, supplemented by histopathological and neuroimaging results, have become the new trends in diagnosing AD (33, 34). Current research on AD has mainly focused on the gene level. Therefore, the search for genetic biomarkers for AD, in combination with amyloid, tau, and microglial PET imaging and genetic marker detection, is important for the comprehensive diagnosis of AD and the identification of potential therapeutic targets for AD (35).

Our study found that hub genes played a role in the differentiation and growth of neural cells and in the transmission of neurotransmitters. These hub genes were mainly enriched in immune/infection pathways (e.g., cholera infection and Helicobacter pylori infection pathways) and other metabolic pathways. Besides, activated mast cells, regulatory T cells, plasma cells, neutrophils, T follicular helper cells, CD8 T cells, resting memory CD4 T cells, and M1 macrophages were the core immune cells contributing to AD progression. The results indicated that immune pathways and immune cells played an important role in the occurrence and development of AD. A growing number of studies have shown the crosstalk between the immune system, neuroinflammation, and pathogenesis of AD, and exploring the underlying mechanisms in detail is very important for the diagnosis of AD (36). Through whole-genome sequencing and whole-exome sequencing, genome-wide association studies have detected several genetic loci associated with AD, including rare immune-related variant genes, and have reported these genes to be enriched in immunity, inflammation, immune protein deposition, and other routes. Many genes associated with AD have been identified, including the whole genome of TR2 and phagocytic receptors. Targeted neuroinflammation *via* CD33 inhibition and/or TREM2 activation may be of considerable importance for neurodegeneration in AD (37). As is well-known, amyloid deposition in the brain is the initiating event in AD (38). Failure of cell-mediated systemic clearance of amyloid leads to extracellular amyloid accumulation, neuroinflammation, oxidative stress, and apoptosis. Altogether, these factors induce AD progression (36). An increase in the levels of inflammatory markers and the identification of AD-associated risk genes associated with innate immune function in AD patients suggest that neuroinflammation is closely related to the pathogenesis of AD (39). Furthermore, neuroinflammation in the microglia, astrocytes, cytokines, chemokines, and complement system also plays an important role in AD. Nonetheless, a set of AD-associated risk genes and immune cells related to immune infiltration has not been screened, and their value in AD assessment has not been explored.

In the present study, data were mined from gene chips extracted from hippocampus brain regions through an autopsy in AD patients and normal individuals. WGCNA was performed to explore changes in gene expression between AD patients and healthy individuals. Compared with other traditional differential expression analysis methods, WGCNA has many advantages. For instance, it analyzes co-expression patterns and reveals functional modules containing related genes. The final screened genes may be used as detection biomarkers or therapeutic targets.

In this study, four potential genes (*ATP2A2, ATP6V1D, CAP2*, and *SYNJ1*) were screened using multiple mechanistic computational methods. By conducting WGCNA to compare the changes in gene expression between AD patients and healthy individuals, the key genes in the module could be used as detection biomarkers or therapeutic targets. We identified nine co-expression modules by dynamic tree cutting, and the genes in the module with the most significant *P*-values were selected for the GO and KEGG analysis and the PPI network analysis (key module; *P* < 0.01). A network was constructed and used to identify the core genes. Five hub genes were identified, namely, *ATP2A2, ATP6V1D, CAP2, SYNJ1*, and *GHITM*. A logistic regression model was constructed and applied to perform a comprehensive evaluation of their ability to predict AD and find other AD datasets (gene expression datasets in hippocampus brain regions) in order to validate the diagnostic value of the model. The intersection genes between the hub genes and blue module were *ATP6V1D, CAP2, SYNJ1*, and *ATP2A2*. GSEA of the core genes was performed to explore the enriched pathways associated with these genes. Several pathways, such as oxidative phosphorylation, citric acid cycle, cholera infection, and *H. pylori* infection pathways, are pivotal for the high expression of AD. Finally, the disease immune infiltration status (i.e., proportion and correlation of immune cells) was assessed (33). Lasso regression was conducted on 22 immune cells to build an immune cell penetration score model. The analysis confirmed the mutual crosstalk between the hub genes, immune microenvironment, and pathogenesis of AD.

Through data mining, we showed that the pathogenesis of AD involved several pathways, the most important being oxidative phosphorylation, citric acid cycle, and immune/infection pathways. The brain requires a constant supply of energy; the majority of such energy is derived from ATP produced by oxidative phosphorylation of glucose in the mitochondria, whereas a small amount of ATP is produced through aerobic glycolysis in the cytoplasm. The phosphorylation pathway and abnormal glucose metabolism complement each other, and abnormal glucose metabolism is closely related to the pathogenesis of AD. A related study reported that impaired glucose levels in specific brain areas preceded the onset of AD symptoms in older patients with neurodegenerative diseases (40). According to a previous relevant report, hypoglycemic metabolism in the brain preceded memory loss and cognitive decline, whereas ketone metabolism in the brain corrected some defects related to glucose hypometabolism through glycolysis (41). In this study, we enriched the citric acid cycle pathway by mining databases, which supports the findings of previous studies. Previous studies showed that oxidative stress was involved in the course of AD and early amnestic mild cognitive impairment (aMCI). Some of these pathways were altered in the aMCI stage (42). We also enriched the oxidative stress pathway through data mining, which is consistent with the results of previous studies. Immune/infection pathways contribute to the pathophysiology of AD. Previously, Nikolic et al. had discovered that the cholera toxin and cholera infection pathways may induce neurodegeneration (43). During aging and neurodegeneration, the immune system activates generated pro-inflammatory mediators (44), possibly leading to a positive feedback loop between neurons and microglia, resulting in persistently low levels of inflammation (45). Nevertheless, there are still several gaps in the study on immune infiltration pathways and the pathogenesis of AD.

Through bioinformatics analysis and calculation, we screened significantly different genes between the AD and normal groups, namely, *ATP2A2, ATP6V1D, CAP2*, and *SYNJ1* (Figure 1). Mutations in *ATP2A2* are thought to cause dyskeratosis and abnormal intercellular adhesion (46), and SERCA plays an important role in Ca^2+^ regulation. Impaired SERCA activity may lead to various diseases, such as AD, diabetes, heart failure, and cancer (47). Previous studies have reported that ATP6V1D is involved in the transport of hydrogen ions and is a core component of vascular ATPase. Currently, there is no relevant literature supporting a relationship between ATP6V1D and AD (48, 49). Adenylate cyclase-associated protein 2 (CAP2) is involved in the regulation of cellular actin dynamics, and CAP2/hap43 regulates the transcription of various genes (50). Previous studies have linked this protein to tumor progression. However, its expression in AD has not yet been evaluated (51). Previous studies have confirmed that SYNJ1 is a lipid phosphatase that is enriched in the brain and is mainly involved in autophagosome/endosomal trafficking, synaptic vesicle recycling, and phosphatidylinositol metabolism. SYNJ1 polymorphism modifies the onset age of AD. Moreover, SYNJ1 is associated with amyloid-induced toxicity. However, the distribution and mechanism of SYNJ1 in the brains of patients are still unclear. Studies have confirmed that SYNJ1 is upregulated in NFTs, plaque-associated dystrophic axons, and Hirano bodies. SYNJ1 immunoreactivity in neurons and senile plaques was elevated in AD patients with one or two APOE ε4 alleles (52). In all types of AD, SYNJ1 contributes to memory deficits in aging hippocampi (53).

Finally, we compared immune cell infiltration between AD patients and controls (Figures 8A,B). Correlation analysis of 22 immune cells showed that the number and proportion of immune cells in the hippocampus of AD patients were more abundant than those in healthy individuals, suggesting that core immune cells, such as regulatory T cells, neutrophils, plasma cells, activated mast cells, T follicular helper cells, CD8 T cells, resting memory CD4 T cells, and M1 macrophages, are involved in promoting AD progression.

Owing to the limited number of samples, confirmation of these preclinical observations will be necessary for future clinical studies of novel biomarkers. A previous study on clinically diagnosed AD identified 25 genome-wide loci, analyzed risk genes and pathways, and identified some rare variants of AD (5). Another study reported that neutrophil counts and neutrophil proportions associated with the lymphocyte ratio were associated with the clinical symptoms, pathological features, and imaging characteristics of AD (54). However, genes related to AD inheritance (such as *ATP6V1D* and *CAP2*) have not been fully explored, and the immune infiltration cells related to AD are still being explored. This study explored the crosstalk between genes and immune cells related to AD, and the analytical results require further clinical and experimental validation.

However, this study has some limitations. First of all, this paper selects the hippocampal dataset from the AD dataset in the GEO database for analysis. The AD dataset in the GEO database is not as large as the tumor dataset, so it should be analyzed and verified with as many datasets as possible to ensure the accuracy of the results. Hippocampal datasets in other databases are also very limited. Combining the data of GEO datasets with other databases will lead to problems, such as batch effect, which is also one of the limitations of this paper. As the hippocampus is the key brain region related to neurogenesis in AD, it was selected as the research object without considering other brain regions related to AD, such as the frontotemporal lobe. Focusing only on the analysis selection of the hippocampus may lead to potential deviation of the analysis results (or the possibility of losing information due to the absence of other brain regions involved in the analysis). Therefore, in future research, we will try to combine multiple brain regions to analyze the transcriptional characteristics of the hippocampus (55). Detailed research needs the support of sequencing results, such as GWSA on larger platforms. Second, in the GEO database, AD has less clinical information; therefore, there is less information that can be used for data analysis. Since this study uses data from public databases, the use of MMSE, MOCA, and other clinical information needs to be approved by the author of the uploaded data, so this part cannot be included. Third, the genes and immune cells screened in this study were limited to data analysis. This study only analyzes the correlation between AD and immune genes. The binding analysis of immune genes and APP / PS1 / PS2 requires further molecular biological experiments, which is also one of the limitations of this study. It is necessary to verify the results of data analysis through *in vivo, in vitro*, and clinical experiments in order to determine whether the screened genes are different between the diseased and normal groups and whether the difference is statistically significant.

## Conclusion

In this study, the comprehensive evaluation is carried out through many mechanical analysis methods. WGCNA, in combination with clinical information, could be applied to screen for the key module in AD and to analyze the biological roles of genes in the modules. Hub genes were screened, and predictive models were constructed. The performance of the predictive model was verified using test and validation sets. The intersection of genes between hub genes and genes in the core module were subjected to GSEA, which revealed a crucial enrichment pathway for the genes in AD. Finally, the immune infiltration results indicated that T follicular helper cells, CD8 T cells, plasma cells, activated mast cells, neutrophils, regulatory T cells, resting memory CD4 T cells, and M1 macrophages were the core immune cells contributing to AD progression. To date, the relationship between immune-related genes and immune-infiltrating cells has not been clearly reported, and the mechanism of their action in the diagnosis of AD needs to be explored.

## Data availability statement

The original contributions presented in the study are included in the article/supplementary material, further inquiries can be directed to the corresponding author.

## Ethics statement

Ethical review and approval was not required for the study on human participants in accordance with the local legislation and institutional requirements. Written informed consent from the patients/participants or patients/participants' legal guardian/next of kin was not required to participate in this study in accordance with the national legislation and the institutional requirements.

## Author contributions

KD, YuaM, QZ, and JT conceived and designed this article. KD is responsible for all the calculations in this paper, draws conclusions from the survey results, including finding and downloading data, normalizing the data, screening differential genes, WGCNA analysis, immune infiltration analysis, writing the manuscript, including summary, introduction, methods, results, and discussion. YuyM contributed to the revision and guidance of the language. All authors read and approved the final manuscript.

## Funding

This work was funded by Major Research Plan of National Natural Science Foundation of China (Grant No. 92163213), General Program of National Natural Science Foundation of China (Grant No. 81970085), Tianjin Science and Technology Plan Project (Grant No. 21JCZDJC00940), Tianjin Health Science and Technology Projects (Grant No. TJWJ2022XK001), and Tianjin Key Medical Discipline (Specialty) Construction Project (Grant No. TJYXZDXK-006A).

## Conflict of interest

The authors declare that the research was conducted in the absence of any commercial or financial relationships that could be construed as a potential conflict of interest.

## Publisher's note

All claims expressed in this article are solely those of the authors and do not necessarily represent those of their affiliated organizations, or those of the publisher, the editors and the reviewers. Any product that may be evaluated in this article, or claim that may be made by its manufacturer, is not guaranteed or endorsed by the publisher.
